# Measurement of health-related quality of life in patients with diabetes mellitus using EQ-5D-5L in Hong Kong, China

**DOI:** 10.1007/s11136-020-02462-0

**Published:** 2020-03-05

**Authors:** Eliza Lai yi Wong, Richard Huan Xu, Annie Wai ling Cheung

**Affiliations:** Jockey Club School of Public Health and Primary Care, The Chinese University of Hong Kong, Hong Kong, China

**Keywords:** Health-related quality of life, Diabetes mellitus, EQ-5D index score, China

## Abstract

**Purpose:**

This study aimed to estimate the health preference scores of the Chinese population with Diabetes Mellitus (DM) using the EQ-5D-5L Hong Kong (HK) population tariff according to different sociodemographic characteristics in HK.

**Methods:**

Data were obtained from a cross-sectional, territory-wide study of patient experience on specialist outpatient services in a public setting in HK. The EQ-5D-5L HK was used to collect the patients’ health status. A total of 2326 respondents were reported to suffer from DM, and their information was elicited and used for the analysis in this study. A robust ANOVA method was used to compare the differences in EQ-5D-5L index scores among subgroups. Binary logistic regressions were used to predict the probability of respondents reporting full health, and ordinal least square (OLS) model was used to assess the relationship between DM and health-related quality of life (HRQoL).

**Results:**

The mean EQ-5D-5L index score for DM patients was 0.84. A total of 229 EQ-5D health states were reported. Altogether, 47.5% of the respondents reported having some problems with pain/discomfort, followed by mobility (26.4%), usual activities (26.0%), and anxiety/depression (23.5%). Logistic regression and OLS models indicated that male and fully employed respondents were less likely to report having problems with any of the five dimensions and index score of EQ-5D than female and non-fully employed respondents. The findings of OLS model also showed that DM patients that experience comorbidity with three and more chronic conditions were more likely to show a lower index score than respondents who reported living with DM alone.

**Conclusion:**

The EQ-5D index scores varied among DM patient characteristics and were more highly impaired with multimorbidity status. Interventions targeting at-risk subgroups, such as modifying single-diseased guidelines, might be helpful to improve their HRQoL.

**Electronic supplementary material:**

The online version of this article (10.1007/s11136-020-02462-0) contains supplementary material, which is available to authorized users.

## Introduction

Diabetes mellitus (DM) is a chronic condition that is due to a lack of insulin from the pancreas or an inadequate efficiency of insulin level leading to severe complications in many parts of the body and significantly increasing the risk of disability and premature death [1]. In the long term, DM is prone to causing heart attack, stroke, kidney failure, leg amputation, vision loss, nerve damage, and even depression [1–5], resulting in negative associations with physical, mental, and social well-being on an individual as well as on health-related quality of life (HRQoL). In a World Health Organization report, DM ranks as the seventh leading cause of deaths globally, with an estimation of 1.6 million direct deaths per year [6]. In Hong Kong (HK), DM has been listed as one of the major causes of morbidity and mortality. In 2017, more than 14,000 inpatient discharges and deaths were related to DM, accounting for 0.6% of total [7].

Given the globally increasing prevalence of DM, strategies to assess the influence of this disease as well as the outcomes of clinical interventions and effectiveness of healthcare policies in different populations are necessary. Currently, clinical indicators are still considered to be the golden criteria for assessing the effectiveness of interventions. However, academic debates on this issue have been raised because clinical indicators are insufficient to capture the overall well-being of an individual with DM [8–10]. Recently, there has been increasing attention on patients’ self-reported health outcomes. Generic preference-based measures (GPBMs) are being utilised to measure the HRQoL of DM patients [11]. Measuring HRQoL can capture the variations in health status of patients with different demographic backgrounds and socioeconomic characteristics at different stages of DM [12]. Quantifying these differences in the health status of DM patients is critical for enabling healthcare professionals to understand the relationship between DM and individuals’ health and well-being. Additionally, GPBMs can provide information on different domains of health and/or well-being for resource allocation by conducting economic evaluation of healthcare polices or clinical interventions and then facilitating decision-making [13, 14].

Currently, the EuroQol five-dimension questionnaire (EQ-5D) is one of the most extensively used GPBMs in measuring health status around the world [15]. This measure can derive an index score based on the local value set to estimate quality-adjusted life years and facilitate a cost–utility analysis [11]. EQ-5D is the preferred measure of HRQoL for health technology assessment in many European countries [16, 17]. Limited cross-sectional studies using GPBMs to explore the relationship between DM and HRQoL among Chinese population have been conducted in HK. Luk and colleagues, using a UK tariff, reported that female, older, and obese DM respondents were more likely to report a lower EQ-5D index score [18]. Wan and colleagues, using SF-6D, found that being female, unmarried, current smoker, and obese were predictors of poor HRQoL among people with DM [19]. Xu and colleagues, using an EQ-5D HK population tariff, indicated that the patients with multimorbidity tend to report a low HRQoL; however, the profile of DM was not specifically reported [20]. Since preference-based HRQoL is strongly influenced by cultural contexts and the local health system design, it is important to study and report the relationship between DM and individuals’ HRQoL using the validated HK EQ-5D tariff. Thus, this study aimed to estimate health preference-based index scores of the Hong Kong Chinese population with DM using the EQ-5D-5L HK population tariff. Such findings could provide an overall summary of HRQoL of individuals with DM, which is a useful reference for the economic evaluation of DM management and an important milestone in the development of patient-centred care.

## Method

### Data collection

Secondary data from a territory-wide cross-sectional patient experience survey among the attendees at specialist outpatient clinics (SOPCs) in HK were collected [21]. Respondents were recruited from all 26 public SOPCs across all 18 districts in HK. The targets were those who used specialist outpatient services during the survey period, aged ≥ 18 years, and able to understand and speak Cantonese. Attendees at paediatric, hospice, psychiatric, dental, anaesthesiology, pathology, or nurse-led or multispecialty outpatient clinics were excluded from the study. Experienced interviewers conducted the telephone survey within 2 weeks after attendance. A structured questionnaire was used in the survey to indicate the attendees’ experience using the HA services. In addition, the sociodemographic characteristics and health status of the respondents were collected regarding self-reported chronic conditions and through the questions of EQ-5D.

### Health preference score using EQ-5D-5L

The EQ-5D-5L HK Chinese version was employed to report the health status and evaluate the health preference score of the respondents. The EQ-5D-5L HK was developed and validated in HK cultural settings based on the international protocol provided by the EuroQol Group, which allowed the evaluation of people’s HRQoL by considering their perceptions of the HK context of culture and value systems [22]. EQ-5D-5L has five dimensions: mobility (MO), self-care (SC), usual activities (UA), pain/discomfort (PD), and anxiety/depression (AD). Each dimension has five levels: no, slight, moderate, severe problems, and extreme/unable to. All health states defined by EQ-5D-5L can be converted into a single health preference index to provide a summary of HRQoL using the HK population tariff [22].

### Statistical analysis

Descriptive statistics were used to summarise respondent characteristics. The health preference score was reported as means and standard deviations (SD). The demographic and socioeconomic characteristic information were recorded for analysis. Age and educational attainment were categorised into three separate groups; living (live alone, live with family/others, live in the institution) and employment status (retired, unemployed, and employed) were used as proxy questions to reflect the respondents’ socioeconomic situation. Multimorbidity with DM was categorised into four groups [1 (only DM), 2 (DM with 1 more chronic disease), 3 (DM with 2 more chronic diseases), and ≥ 4 (DM with 3 or more chronic diseases)]. A chi-squared test was used to compare the differences in the reporting problems on each dimension of EQ-5D-5L across different subgroups. Given that the EQ-5D-5L index score was non-normally distributed (Shapiro–Wilk test, *p* < 0.05), the differences in EQ-5D-5L index scores among subgroups were assessed using the bootstrap version (*n* = 599) of a robust ANOVA method [23]. Binary multivariable logistic regressions were used to predict the probability of respondents reporting full health (0 and 1, where 0 indicates no problem and 1 indicates any problem reported) on each of the five dimensions of EQ-5D-5L. Ordinal least squares (OLS) model was used to explore the relationship between DM and EQ-5D index score. Pairwise deletion was performed to resolve missing values. The data were analysed using R (R foundation, Austria) and STATA (StataCorp LP, TX, USA), and statistical significance was set as *p* value ≤ 0.05.

## Results

A total of 13,966 respondents completed the survey, of which 2326 who suffered from DM were extracted for secondary data analysis. The demographics of the extracted study population is shown in Table 1. Nearly half of the respondents were female (50%) and retired (56.3%). The majority of them were aged ≥ 65 years (60%), only had a primary educational level or below (52%), were living with families (93%), and self-reported more than one chronic disease (70%).

**Table 1:** Characteristics of the respondents reported with DM (*n* = 2326)

	Overall	
	*n*	%
Sex		
Male	1143	49.1
Female	1183	50.9
Age group (mean [sd])		
18–44	73	3.1
45–64	876	37.7
≥ 65	1377	59.2
Education		
No/Primary	1208	52.0
Secondary/Post-secondary	912	39.2
Tertiary or above	204	8.8
Current living status		
Live alone	148	6.4
Live with family/others	2161	93.1
Live in institution^a^	12	0.5
Current work status		
Retired	1307	56.3
Unemployed^b^	444	19.1
Employed^c^	572	24.6
Multimorbidity		
DM only [1]	725	31.2
DM with 1 more CD [2]	1071	46.0
DM with 2 more CD [3]	508	21.8
DM with 3/ more CD [≥ 4]	22	0.9

*CD* chronic disease(s)

^a^Include Convalescent Hospital/Rehabilitation Hospital/Hospital, and old age home

^b^Unemployment included unemployed, home-maker and full-time student

^c^Employment included full-time worker and part-time worker

**Fig. 1:**
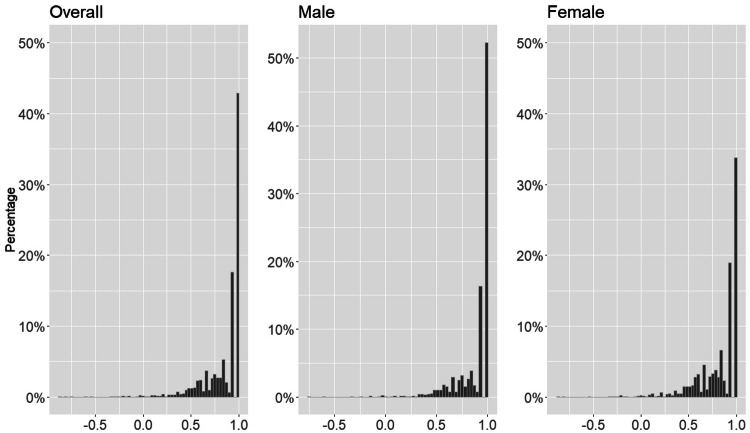
The distribution of EQ-5D index scores for overall, and stratified by male and female

Overall, the mean health preference-based index score using EQ-5D-5L in respondents with DM was 0.84 out of 1 with a range between -0.86 and 1.0. Table 2 presents the estimated health preference-based score of respondents with DM by demographics, socioeconomic status, and level of multimorbidity. Among respondents with DM, 42.9% reported full health (index score = 1.0). Men seemed to have better health conditions than women; 52.3% of men reported full health vs. only 33.8% of women. Both male and female respondents had higher health preference-based index scores if they were young, were highly educated, lived with their families, were fully employed, and suffered from few chronic conditions. Figure 1 depicts the distribution of EQ-5D-5L index scores; the scores were highly skewed (> 40% reported full health).

**Table 2:** The EQ-5D index score of the respondents reported without and with DM (stratified by sex)

	Overall		Male		Female	
	Mean (SD)	*p* value	Mean (SD)	*p* value	Mean (SD)	*p* value
Overall						
EQ-5D index score	0.84 (0.23)		0.88 (0.2)		0.81 (0.24)	< 0.01
EQ-5D index score = 1, %	42.9		52.3		33.8	
Range of index score	− 0.86 to 1.0		− 0.76 to 1.0		− 0.86 to 1.0	
Age group						
18–44	0.95 (0.1)	< 0.001	0.93 (0.12)	< 0.001	0.96 (0.08)	< 0.001
45–64	0.89 (0.17)		0.92 (0.15)		0.87 (0.17)	
≥ 65	0.80 (0.26)		0.85 (0.23)		0.76 (0.27)	
Education						
No/Primary	0.8 (0.25)	< 0.001	0.85 (0.22)	0.001	0.77 (0.27)	< 0.001
Secondary/ Post-secondary	0.88 (0.19)		0.90 (0.2)		0.86 (0.18)	
Tertiary or above	0.89 (0.13)		0.90 (0.15)		0.87 (0.15)	
Current living status						
Live alone	0.79 (0.23)	0.014	0.86 (0.21)	0.105	0.75 (0.24)	0.359
Live with family/others	0.85 (0.22)		0.88 (0.2)		0.82 (0.23)	
Live in institution ^a^	0.45 (0.57)		0.61 (0.23)		0.33 (0.72)	
Current work status						
Retired	0.8 (0.26)	< 0.001	0.84 (0.23)	0.001	0.74 (0.29)	< 0.001
Unemployed^b^	0.85 (0.19)		0.81 (0.24)		0.86 (0.18)	
Employed^c^	0.94 (0.11)		0.94 (0.11)		0.92 (0.1)	
Multimorbidity						
1	0.88 (0.19)	< 0.001	0.91 (0.18)	< 0.001	0.86 (0.2)	< 0.001
2	0.83 (0.24)		0.88 (0.19)		0.79 (0.27)	
3	0.81 (0.24)		0.84 (0.24)		0.78 (0.24)	
≥ 4	0.69 (0.3)		0.68 (0.36)		0.71 (0.2)	

*EQ-5D* EuroQol five-dimension five levels, *SD* standard deviation

^#^The comparison of index score between male and female

^a^Include Convalescent Hospital/Rehabilitation Hospital/Hospital, and old age home

^b^Unemployment included unemployed, home-maker and full-time student

^c^Employment included full-time worker and part-time worker

Table 3 presents the most frequently reported health states among all the 229 health states and the corresponding index score for participants with DM. The full health state was ‘11,111′, and nearly 15% of the respondents (*n* = 339) reported a health state of ‘11,121′ (index score = 0.924), which indicates that the respondents reported no problems on MO, SC, UA, and AD, but had slight problems on PD. Among the top 32 health states, the lowest index score was ‘22,332' (*n* = 11, index score = 0.482), signifying a slight problem on MO, SC, and AD and a moderate problem on UA and PD. The health states reported by female respondents were similar to those in the overall pattern; however, for male participants, the order of health states was slightly different. Distributions according to sex are reported in the Online Appendix.

**Table 3:** The most frequent reported health states of EQ-5D among the respondents

	State	*n*	%	%%^d^	Index score
1	11,111^a^	997	42.86	42.86	1.0
2	11,121	339	14.57	57.43	0.924
3	11,122	73	3.14	60.57	0.844
4	11,112	57	2.45	63.02	0.919
5	11,131	44	1.89	64.91	0.852
6	21,221	41	1.76	66.67	0.747
7	11,221	33	1.42	68.09	0.857
8	21,222	33	1.42	69.51	0.667
9	22,222	31	1.33	70.84	0.581
10	11,132	29	1.25	72.09	0.772
11	11,133	25	1.07	73.16	0.712
12	21,211	25	1.07	74.23	0.823
13	21,121	23	0.99	75.22	0.815
14	11,222	20	0.86	76.08	0.777
15	22,211	19	0.82	76.90	0.736
16	22,221	19	0.82	77.72	0.661
17	11,211	15	0.64	78.36	0.932
18	22,322	15	0.64	79.00	0.554
19	11,113	14	0.60	79.60	0.860
20	22,231	13	0.56	80.16	0.589
21	21,111	12	0.52	80.68	0.890
22	21,122	12	0.52	81.20	0.734
23	21,231	12	0.52	81.72	0.676
24	22,232	11	0.47	82.19	0.509
25	22,332	11	0.47	82.66	0.482
26	32,221	11	0.47	83.13	0.588
27	21,232	9	0.39	83.52	0.596
28	11,123	8	0.34	83.86	0.784
29	31,311	8	0.34	84.20	0.723
30	11,142	7	0.30	84.50	0.612
31	22,233^b^	7	0.30	84.80	0.449
32	31,111	7	0.30	85.10	0.817
	⁝	⁝	⁝	⁝	⁝
	55,555^c^	1	0.04	100.00	− 0.864

^a^11111 means the respondents choose no problem on all five dimensions of EQ-5D-5L descriptive system

^b^22233 means the respondents choose slight problem on mobility, self-care and usual activities; moderate problem on pain/discomfort and anxiety/depression on EQ-5D-5L descriptive system

^c^Overall, 229 states were reported

^d^%% cumulative percentage

Table 4 demonstrates the proportion of the respondents reporting having ‘any problem’ on each dimension of EQ-5D-5L. The percentages of having any problem on the dimensions PD, MO, UA, AD, and SC were 47.5%, 26.4%, 26.0%, 23.5%, and 14.1%, respectively. The proportion of reporting ‘any problems’ was higher in the subgroups of women, those with low educational levels and living in the institute, and those who were retired or with multimorbidity than that in the other subgroups across the five dimensions of the EQ-5D-5L.

**Table 4:** Proportion of the respondents reported any problems on the five dimensions of EQ-5D-5L

	Mobility		Self-care		Usual activities		Pain/discomfort		Anxiety/depression	
	%	*p* value	%	*p* value	%	*p* value	%	*p* value	%	*p* value
Overall	26.4		14.1		26.0		47.5		23.5	
Sex										
Male	21.5	< 0.001	11.4	< 0.001	20.2	< 0.001	38.4	< 0.001	17.7	< 0.001
Female	31.3		16.7		31.6		56.2		29.2	
Age group										
18–44	4.1	< 0.001	1.4	< 0.001	5.5	< 0.001	28.8	< 0.001	16.4	0.11
45–64	14.2		5.9		14.4		42.0		22.0	
≥ 65	35.4		20.0		34.5		51.9		24.8	
Education										
No/Primary	34.8	< 0.001	20.0	< 0.001	34.7	< 0.001	53.9	< 0.001	26.5	0.002
Secondary/Post-secondary	19.1		7.7		17.6		40.8		20.0	
Tertiary or above	16.9		7.8		16.3		39.7		22.1	
Current living status										
Live alone	35.1	< 0.001	16.2	< 0.001	31.1	< 0.001	56.1	< 0.001	33.1	0.02
Live with family/others	25.3		13.6		25.3		46.7		22.9	
Live in institution	91.7		58.3		75.0		83.3		16.7	
Current work status										
Retired	36.1	< 0.001	21.1	< 0.001	34.9	< 0.001	51.9	< 0.001	25.2	< 0.001
Unemployed	23.6		9.2		24.5		54.5		27.9	
Employed	6.5		1.9		7.0		32.0		16.3	
Multimorbidity										
1	21.3	< 0.001	20.7	< 0.001	22.5	< 0.001	27.5	0.001	27.1	< 0.001
2	49.7		46.6		48.3		47.4		46.6	
3	27.5		30.5		27.6		23.8		23.8	
≥ 4	1.5		2.1		1.7		1.3		2.6	

*EQ-5D* EuroQol five-dimension five levels, *SD* standard deviation, *MO* mobility, *SC* self-care, *UA* usual activities, *PD* pain/discomfort, *AD* anxiety/depression

% Percentage of reported any problem on dimension

The binary logistic regression models indicated that it is highly possible for female DM patients to report having health problems on all the dimensions of EQ-5D. Compared with the respondents living alone, living in the institute was a statistically significant determinant for reporting problems in the MO dimension. Age had an impact on all the dimensions except for AD dimension. Additionally, DM patients living with three and more chronic conditions tended to report having more problems [Odds ratio (OR) 7.52, 95% CI 3.02–20.43) on AD as well as the SC dimension (OR 5.13, 95% CI 1.81–13.51) than patients reported living with DM alone (Table 5).

**Table 5:** Binary multivariate logistic and OLS regression of responses to the EQ-5D for overall

	Mobility		Self-care		Usual activities		Pain/discomfort		Anxiety/depression		Index score	
	OR	95% CI	OR	95% CI	OR	95% CI	OR	95% CI	OR	95% CI	beta	95% CI
Female	1.42**	**1.13, 1.78**	1.43*	**1.09,1.89**	1.6*	**1.28,2.01**	1.81***	**1.5,2.18**	1.79***	**1.44, 2.24**	− 0.058***	** − 0.077, − 0.039**
Age	1.07***	**1.05, 1.08**	1.08***	**1.06,1.09**	1.06***	**1.05,1.08**	1.01*	**1,1.02**	1	0.99, 1.01	− 0.004***	** − 0.005, − 0.003**
Secondary/Post-secondary	0.83	0.65, 1.06	0.8	0.58,1.09	0.78	0.61,0.99	0.84	0.69,1.02	0.85	0.67, 1.07	0.018	** − **0.002, 0.038
Tertiary or above	0.89	0.58, 1.32	0.67	0.37,1.14	0.8	0.52,1.2	0.85	0.61,1.17	1.02	0.69, 1.48	0.021	** − **0.012, 0.054
Live with family/others	0.76	0.52, 1.13	1.06	0.66,1.77	0.95	0.65,1.43	0.78	0.55,1.11	0.66*	0.46, 0.97	0.033	** − **0.003, 0.069
Live in institution	11.02*	**1.89, 21.35**	3.26	0.89,12.92	3.41	0.9,16.92	2.94	0.73,19.75	0.36	0.05, 1.44	− 0.277***	** − 0.401, − 0.152**
Unemployed	1.04	0.76, 1.42	0.78	0.51,1.17	1.07	0.79,1.46	1.06	0.81,1.38	0.98	0.73, 1.32	0.016	** − **0.012, 0.043
Employed	0.46***	**0.3, 0.69**	0.34**	**0.17,0.65**	0.53**	**0.35,0.79**	0.67**	**0.5,0.88**	0.71*	**0.5, 0.99**	0.031*	**0.003, 0.06**
Multimorbidity												
2	1.37*	**1.07, 1.76**	1.18	0.85,1.63	1.22	0.95,1.56	1.14	0.93,1.39	1.11	0.88, 1.41	** − **0.02	** − **0.04, 0
3	1.55**	**1.17, 2.07**	1.56*	**1.09,2.24**	1.49*	**1.12,1.98**	1.31*	**1.03,1.66**	1.29	0.97, 1.7	− 0.035**	** − 0.06, − 0.011**
≥ 4	2.53	0.95,6.4	5.13**	**1.81,13.51**	3.11**	**1.2,7.79**	2.52	1.02,6.8	7.52***	**3.02, 20.43**	− 0.173***	** − 0.264, − 0.081**

Reference: Male, no/primary educational level, live alone, retired, and no multimorbidity

Bold values are statistically significant

*95% CI* 95% confidence interval, *OR* odds ratio

*p < 0.05; **p < 0.01; ***p < 0.001

Table 5 showed that the DM patients who were female (− 0.058), old (− 0.004) and living in the institute (− 0.227) were more likely to show worse HRQoL (low index score) than the other DM patients. Moreover, the fully employed status had a positive impact on HRQoL for DM patients (beta = 0.031, 95% CI 0.003–0.06). After adjusting for socioeconomic and demographic factors, our model identified that the respondents living with DM and two additional chronic conditions were more likely to show a lower index score than the patients (beta =  − 0.035, 95% CI − 0.06 − 0.011) living with DM alone. The relationship was even more negative when the DM patients reported living with more than two chronic conditions (beta =  − 0.173, 95% CI − 0.264 to −  0.081). We found no statistically significant effect of educational attainment in the regression model.

## Discussion

This study is first of its kind to employ secondary data from a territory-wide population survey in HK with the locally validated EQ-5D-5L instrument to report the health preference-based index score for DM patients. The findings show that the mean of health preference-based index scores for individuals with DM was 0.84, which is comparatively lower than the index score of 0.92 in the general population [24]. Among DM patients, women reported lower index scores than did men, and the difference still appeared to be statistically significant even after adjusting for socioeconomic factors. The most obvious enhancement in the EQ-5D-5L index score was observed among fully employed patients. Age seems to have a relationship with decreasing HRQoL among all the DM patients. However, no statistically significant difference was identified in the EQ-5D-5L index scores among respondents with different educational levels, which was slightly different from findings from a previous study [25, 26]. A Spanish study reported that patients reporting having DM had a mean EQ-5D-5L index score of 0.742, and women scored lower than men [27]. McClure and colleagues reported that the mean index score was 0.790 for DM patients in Canada [28]. However, a recent study conducted in Finland shows that the mean EQ-5D-5L index score was 0.85 among the respondents with DM [29], which is similar to our results. When comparing our findings with those from other Asian countries, the mean index score in HK is a bit lower than in Korea, 0.87 [30], Japan, 0.86 [31], Singapore, 0.85 [32], and 0.87 in East China [33].

Our results show that DM patients with multimorbidity status had statistically significant lower EQ-5D-5L index scores than patients with DM alone. This is in line with the other studies [31–33]. A study conducted in China on DM patients reported that the mean EQ-5D-5L index score is 0.876, which decreased to 0.834 when patients report having comorbidities [33]. A UK study also indicated that patients with multimorbidity diabetes have a lower quality of life than other people [34]. Another study in Singapore reported a decrease in mean EQ-5D-5L index scores of between 0.028 and 0.043 for DM patients who reported having at least two complications [35].

We observed that the reduction in the index scores is strongly associated with having problems in the pain dimension (PD) in DM patients. Among the 10 most frequently reported EQ-5D health states from the respondents, eight included different levels of health problems in the PD dimension. These findings were in line with previous studies in other regions. A study conducted in Thailand reports that, compared to other dimensions of the EQ-5D, more than 50% of DM patients have reported problem with PD [26]. Another study in Singapore shows that 43%, 41%, and 48% of English, Chinese, and Malay speaking DM patients reported problems on PD, respectively [13]. Kapur identified that pain, particularly chronic pain, affects people with DM and interferes with their daily activities [36]. Geelen and colleagues indicated that pain intensity was associated with diminished quality of life [37].

In addition, although there is robust evidence that individuals experience symptoms of anxiety when they are diagnosed with diabetes [38, 39], in our study, DM patients with comorbidities with three or more chronic conditions were seven times more likely to report having problems with anxiety or depression, which was much higher than the findings of a previous study in HK [18]. One study supported the findings that the effect of depression on quality of life is greater than the effect of diabetes on quality of life [39]. Several studies also addressed the idea that DM could increase the probability of suffering anxiety for DM patients in both local and international settings [37, 40]. The effect of multimorbidity on depression among DM patients did not accumulate, wherein the severity of depression might increase with an increased number of comorbidities. Furthermore, although there is a mixed picture of whether EQ-5D is able to reflect some variations of mental problems [41], our study provides some information that EQ-5D seems sensitive to detect the anxiety/depression in the DM Chinese population. Further tests are needed in the field of mental health.

This study provides a reference of health preference scores of the Chinese population with DM using the EQ-5D-5L HK population tariff with different sociodemographic characteristics. The results are important for providing information for future cost–utility analyses of new drugs or policies targeting improving the health outcomes of DM treatments and facilitating the DM service planning at regional, national, and international levels. Moreover, to overcome the ceiling effect and some other characteristics of the EQ-5D data, different regression models (see Online Appendix) were run to ensure the validity and robustness of the estimation in the exploration of the relationship between DM and the HRQoL. The OLS model was proven to perform better than the other methods; however, further studies using other methods to test different populations are needed.

There are several limitations in this study. First, the information about health conditions was based on respondents’ self-reports and we were not able to differentiate the type of DM and comprehensiveness of chronic conditions, which may hinder the subgroup analysis of relationship between DM and HRQoL. Second. The results of HRQoL were only based on the respondents from SOPCs in the public healthcare setting, and there is a lack of respondents with mild stages of DM or those from private settings, which may lead to potential selection bias.

## Conclusions

The relationship between DM and patients’ HRQoL in HK, China, was estimated using the EQ-5D-5L HK. To strive for the development of patient-centred care, the disease group information may provide insight into disease-based variations on HRQoL from a general population approach. Thus, it is important to adopt both generic and disease-specific tools for patient-reported outcomes. We showed that the health preference-based index score varied among DM patient characteristics and were impaired with multimorbidity status. These findings provide a good base for an evaluation of DM service programmes, and future studies are required to determine whether these estimates are consistent in patients with different types of DM or from other clinical settings.

## Electronic supplementary material

Below is the link to the electronic supplementary material.

Electronic supplementary material 1 (DOCX 43 kb)

## References

[1] Solli O, Stavem K, Kristiansen I (2010). Health-related quality of life in diabetes: The associations of complications with EQ-5D scores. Health and Quality of Life Outcomes.

[2] Basaraba R, Ackart D, Lakey N (2015). Hyperglycemia and impaired glucose tolerance exacerbates tuberculosis and diabetes disease severity. Diabetes.

[3] Nambam B, Haller M, Schatz D (2015). Is pancreas volume a marker of type 1 diabetes disease progression? A preliminary report. Diabetes.

[4] Jin B, Liu R, Hao S (2017). Defining and characterizing the critical transition state prior to the type 2 diabetes disease. PLoS ONE.

[5] Pagano G, Polychronis S, Wilson H (2018). Diabetes mellitus and Parkinson disease. Neurology.

[6] World Health Organization. WHO Diabetes. (2018). https://www.who.int/en/news-room/fact-sheets/detail/diabetes.

[7] Centre for Health Protection. Diabetes Mellitus. (2017). https://www.healthyhk.gov.hk/phisweb/en/healthy_facts/disease_burden/major_causes_death/diabetes_mellitus/.

[8] Murillo M, Bel J, Pérez J (2017). Impact of monitoring health-related quality of life in clinical practice in children with type 1 diabetes mellitus. Quality of Life Research.

[9] Weatherall J, Polonsky WH, Lanar S (2018). When insulin degludec enhances quality of life in patients with type 2 diabetes: A qualitative investigation. Health and Quality of Life Outcomes.

[10] Tang TS, Yusuf FLA, Polonsky WH (2017). Assessing quality of life in diabetes: II – Deconstructing measures into a simple framework. Diabetes Research and Clinical Practice.

[11] Sullivan PW, Ghushchyan VH (2016). EQ-5D scores for diabetes-related comorbidities. Value in Health.

[12] Janssen M, Lubetkin EI, Sekhobo JP (2011). The use of the EQ-5D preference-based health status measure in adults with Type 2 diabetes mellitus. Diabetic Medicine.

[13] Wang Y, Tan N-C, Tay E-G (2015). Cross-cultural measurement equivalence of the 5-level EQ-5D (EQ-5D-5L) in patients with type 2 diabetes mellitus in Singapore. Health and Quality of Life Outcomes.

[14] Redekop WK, Koopmanschap MA, Stolk RP (2002). Health-related quality of life and treatment satisfaction in Dutch patients with type 2 diabetes. Diabetes Care.

[15] Herdman M, Gudex C, Lloyd A (2011). Development and preliminary testing of the new five-level version of EQ-5D (EQ-5D-5L). Quality of Life Research.

[16] Rencz F, Gulácsi L, Drummond M (2016). EQ-5D in Central and Eastern Europe: 2000–2015. Quality of Life Research.

[17] Rowen D, Azzabi Zouraq I, Chevrou-Severac H (2017). International regulations and recommendations for utility data for health technology assessment. Pharmacoeconomics.

[18] Luk A, Li X, Ozaki R (2014). Comorbidities, health-related quality of life, and glycemic control in chinese patients with type 2 diabetes: The Hong Kong Joint Asia Diabetes Evaluation (JADE) Program. Diabetes.

[19] Wan E, Fung C, Choi E (2016). Main predictors in health-related quality of life in Chinese patients with type 2 diabetes mellitus. Quality of Life Research.

[20] Wong E, Xu R, Cheung A (2019). Measuring the impact of chronic conditions and associated multimorbidity on health- related quality of life in the general population in Hong Kong SAR, China: A cross-sectional study. PLoS ONE.

[21] Xu RH, Wong ELY (2017). Involvement in shared decision-making for patients in public specialist outpatient clinics in Hong Kong. Patient Preference and Adherence.

[22] Wong ELY, Ramos-Goñi JM, Cheung AWL (2018). Assessing the use of a feedback module to model EQ-5D-5L health states values in Hong Kong. Patient.

[23] Wilcox RR (2011). Introduction to robust estimation and hypothesis testing.

[24] Wong ELY, Cheung AWL, Wong AYK (2019). Normative profile of health-related quality of life for Hong Kong general population using preference-based instrument EQ-5D-5L. Value in Health.

[25] Arifin B, Idrus L, Asselt A (2019). Health-related quality of life in Indonesian type 2 diabetes mellitus outpatients measured with the Bahasa version of EQ-5D. Quality of Life Research.

[26] Pattanaphesaj J, Thavorncharoensap M (2015). Measurement properties of the EQ-5D-5L compared to EQ-5D-3L in the Thai diabetes patients. Health and Quality of Life Outcomes.

[27] Collado Mateo D, García Gordillo MA, Olivares PR (2015). Normative values of EQ-5D-5L for diabetes patients from Spain. Nutricion Hospitalaria.

[28] Mcclure NS, Al Sayah F, Ohinmaa A (2018). Minimally important difference of the EQ-5D-5l index score in adults with type 2 diabetes. Value in Health.

[29] Jalkanen K, Aarnio E, Lavikainen P (2019). Impact of type 2 diabetes treated with non-insulin medication and number of diabetes-coexisting diseases on EQ-5D-5 L index scores in the Finnish population. Health and Quality of Life Outcomes.

[30] Choi YJ, Lee MS, An SY (2011). The relationship between diabetes mellitus and health-related quality of life in Korean adults: The Fourth Korea National Health and Nutrition Examination Survey (2007–2009). Diabetes & Metabolism Journal.

[31] Sakamaki H, Ikeda S, Ikegami N (2006). Measurement of HRQL using EQ-5D in patients with type 2 diabetes mellitus in Japan. Value in Health.

[32] Pan C-W, Sun H-P, Wang X (2015). The EQ-5D-5L index score is more discriminative than the EQ-5D-3L index score in diabetes patients. Quality of Life Research.

[33] Pan C, Sun H, Zhou H (2016). Valuing health-related quality of life in type 2 diabetes patients in China. Medical Decision Making.

[34] Mujica-Mota R, Roberts M, Abel G (2015). Common patterns of morbidity and multi-morbidity and their impact on health-related quality of life: Evidence from a national survey. Quality of Life Research.

[35] Wang P, Luo N, Tai ES (2016). The EQ-5D-5L is more discriminative than the EQ-5D-3L in patients with diabetes in Singapore. Value in Health Regional Issues.

[36] Kapur D (2003). Neuropathic pain and diabetes. Diabetes/Metabolism Research and Reviews.

[37] Geelen CC, Smeets RJEM, Schmitz S (2017). Anxiety affects disability and quality of life in patients with painful diabetic neuropathy. European Journal of Pain.

[38] Egede LE, Ellis C (2010). Diabetes and depression: Global perspectives. Diabetes Research and Clinical Practice.

[39] Goldney RD, Phillips PJ, Fisher LJ (2004). Diabetes, depression, and quality of life: A population study. Diabetes Care.

[40] Lee S, Chiu A, Tsang A (2006). Treatment-related stresses and anxiety-depressive symptoms among Chinese outpatients with type 2 diabetes mellitus in Hong Kong. Diabetes Research and Clinical Practice.

[41] Brazier J (2010). Is the EQ-5D fit for purpose in mental health?. British Journal of Psychiatry.

