# Supplementary material for: Measurement of health-related quality of life in patients with diabetes mellitus using EQ-5D-5L in Hong Kong, China
Source: Qual Life Res. 2020 Mar 5;29(7):1913–21. doi: 10.1007/s11136-020-02462-0 (PMC7295714; doi:10.1007/s11136-020-02462-0)
Supplement: Supplementary file 1 — Electronic supplementary material 1 (DOCX 43 kb) [file 11136_2020_2462_MOESM1_ESM.docx]

**Appendix**

Except for the OLS model, another seven regression models were used in this study. The generalized linear model (GLM), a flexible generalization of OLS that allows for the outcome variables to have a non-normal error distribution, was adopted (the 1 − index score [known as disutility] was used as the outcome variable to avoid the negative values in the GLM model). Moreover, given the EQ-5D index data were censored (-0.8637 ~ 1 in this study), the OLS and GLM may have produced some bias in estimation. Accordingly, the Tobit regression model was adopted. This model is censored and designed to estimate the linear relationships amongst variables when either left- or right-censoring occurs in the dependent variable. A two-part model was also adopted to estimate the differences of index scores for different patient subgroups. It was composed of a logistic regression model, which was used to predict the probability of respondents reporting full health (0 [not full health] and 1 [full health]), and a truncated OLS model, which was used to predict the probability of the respondents reporting non-full health (index score ≠ 1.0). The fifth method was the betamix, which is a robust two-part model. It consists a multinomial logit model and a beta mixture model. The betamix model ensured the beta mixture model to deal with the continuous data between the boundaries and allows the multinomial logit model to deal with the data at the boundaries (observations at full health). Considering there is always a large gap between the mass at one and the next feasible EQ-5D value, the adjusted limited dependent variable mixture model (aldvmm) was also used in the study. In order to simplify the model, only 1-component aldvmm was applied. Another adopted model was logistic quantile regression (LQR) model. The LQR is a robust model, which can give an overall assessment of the covariate effects at different quantiles (median was used in this study) of the outcome rather than the mean. The last model was censored least absolute deviation (CLAD) model. Unlike the Tobit model, the CLAD does not require the assumptions of normality and homoscedasticity. It is appropriate for dealing with the right censoring data. For the selection of the best fit model for the final presentation, the root mean square error (RMSE) based on 10-fold cross validation was used to assess the overall performance of the models. A small value represents a small error (i.e. a good model).

Table A-1 the RMSE of each model

|  | **OLS** | **GLM** | **Tobit** | **Tow-part** | **betamix** | **aldvmm** | **lqr** | **clad** |
| --- | --- | --- | --- | --- | --- | --- | --- | --- |
| RMSE | ***0.210*** | 0.308 | 0.246 | 0.211 | 0.216 | 0.211 | 0.820 | 0.226 |

RMSE, root mean square error

Table A-2 OLS, Tobit regression for EQ-5D index score

|  | **GLM** | |  | **Tobit model** | |
| --- | --- | --- | --- | --- | --- |
|  | *beta* | *95% C.I.* |  | *beta* | *95% C.I.* |
| Female | **0.173***** | **0.094,0.251** |  | **-0.104***** | **-0.134,-0.074** |
| Age | **0.014***** | **0.009,0.017** |  | **-0.006***** | **-0.008,-0.005** |
| Secondary/Post-secondary | -0.075 | -0.168,0.017 |  | **0.036*** | **0.004,0.068** |
| Tertiary or above | -0.121 | -0.292,0.050 |  | 0.021 | -0.031,0.074 |
| Live with family/others | -0.067 | -0.168,0.034 |  | **0.059*** | **0.005,0.113** |
| Live in institution | 0.058 | -0.094,0.210 |  | **-0.208*** | **-0.39,-0.027** |
| Unemployed | -0.029 | -0.138,0.081 |  | 0.008 | -0.035,0.05 |
| Employed | **-0.673***** | **-0.881,-0.463** |  | **0.077**** | **0.031,0.122** |
| Multimorbidity |  |  |  |  |  |
| 2 | **0.096*** | **0.001,0.190** |  | **-0.038*** | **-0.07,-0.006** |
| 3 | **0.142**** | **0.041,0.243** |  | **-0.069***** | **-0.108,-0.031** |
| ≥4 | **0.417***** | **0.220,0.613** |  | **-0.240***** | **-0.376,-0.104** |
|  |  |  |  |  |  |
| RMSE | 0.308 |  |  | 0.246 |  |

* P<0.05, ** p<0.01, *** p<0.001

Reference: Male, no/primary educational level, live alone, retired, and no multimorbidity

95%C.I., 95% confidence interval;RMSE, root mean square error

Table A-3 the result of two-part model

|  | beta. | p-value\| | 95% C.I. | |
| --- | --- | --- | --- | --- |
| ***Logistic part*** |  |  |  |  |
| Female | -0.06724 | 0.72 | -0.43491 | 0.300425 |
| Age | **-0.03963** | **0.002** | -0.06465 | -0.01462 |
| Secondary/post | 0.191928 | 0.45 | -0.30563 | 0.68949 |
| Live with family/others | -0.32149 | 0.421 | -1.10414 | 0.461171 |
| Live in institution | -1.0798 | 0.076 | -2.27454 | 0.114942 |
| Multimorbidity |  |  |  |  |
| 2 | -0.40895 | 0.103 | -0.9008 | 0.082893 |
| 3 | -0.04666 | 0.877 | -0.63561 | 0.542296 |
| ≥4 | -1.10813 | 0.071 | -2.311 | 0.094743 |
|  |  |  |  |  |
| _cons | 5.650864 | 0 | 3.448257 | 7.853472 |
|  |  |  |  |  |
| ***OLS part*** |  |  |  |  |
| Female | **-0.05326** | **0** | -0.06985 | -0.03666 |
| Age | **-0.00343** | **0** | -0.00436 | -0.00251 |
| Secondary/post | 0.015788 | 0.078 | -0.00175 | 0.033323 |
| Tertiary | 0.012663 | 0.379 | -0.01556 | 0.040882 |
| Live with family/others | 0.042986 | 0.006 | 0.012174 | 0.073799 |
| Live in institution | -0.06661 | 0.264 | -0.18339 | 0.050164 |
| Unemployed | 0.004086 | 0.735 | -0.01953 | 0.027699 |
| Employed | 0.030288 | 0.014 | 0.006031 | 0.054544 |
| Multimorbidity |  |  |  |  |
| 2 | -0.01271 | 0.154 | -0.03019 | 0.004772 |
| 3 | **-0.04009** | **0** | -0.06124 | -0.01893 |
| ≥4 | **-0.13134** | **0.001** | -0.21219 | -0.05048 |
|  |  |  |  |  |
| RMSE | 0.211 |  |  |  |

Reference: Male, no/primary educational level, live alone, retired, and no multimorbidity

95%C.I., 95% confidence interval; RMSE, root mean square error

Table A-4 the result of betamix model

|  | beta | p-value | 95% C.I. | |
| --- | --- | --- | --- | --- |
| Female | **-0.34441** | **0** | -0.4405418 | -0.24829 |
| Age | **-0.01693** | **0** | -0.0224952 | -0.01136 |
| Secondary/post | 0.093249 | 0.07 | -0.0074661 | 0.193964 |
| Tertiary | 0.028057 | 0.734 | -0.1335719 | 0.189686 |
| Live with family/others | 0.146431 | 0.108 | -0.0323251 | 0.325188 |
| Live in institution | **-1.22062** | **0** | -1.884614 | -0.55662 |
| Unemployed | 0.013419 | 0.846 | -0.1219825 | 0.148821 |
| Employed | 0.078452 | 0.266 | -0.0596784 | 0.216583 |
| multimorbidity |  |  |  |  |
| 2 | **-0.1029** | **0.044** | -0.2031239 | -0.00268 |
| 3 | **-0.17406** | **0.005** | -0.295952 | -0.05216 |
| ≥4 | **-0.67504** | **0.004** | -1.137607 | -0.21248 |
|  |  |  |  |  |
| _cons | 3.734169 | 0 | 3.261649 | 4.206689 |
| C1_lnphi |  |  |  |  |
| _cons | 0.816678 | 0 | 0.7332169 | 0.90014 |
| C1_phi | 2.26297 |  | 2.081767 | 2.459946 |
|  |  |  |  |  |
| RMSE | 0.216 |  |  |  |

Reference: Male, no/primary educational level, live alone, retired, and no multimorbidity

95%C.I., 95% confidence interval; RMSE, root mean square error

Table A-5 the results of the aldvmm

|  | beta | p-value | 95% C.I. | |
| --- | --- | --- | --- | --- |
| Female | **-0.10789** | **0** | -0.13927 | -0.0765 |
| Age | **-0.00648** | **0** | -0.00824 | -0.00472 |
| Secondary/post | **0.034562** | **0.042** | 0.001273 | 0.067851 |
| Tertiary | 0.021432 | 0.44 | -0.03298 | 0.075845 |
| Live with family/others | **0.056924** | **0.049** | 0.000331 | 0.113518 |
| Live in institution | **-0.30654** | **0.001** | -0.49452 | -0.11856 |
| Unemployed | 0.009642 | 0.669 | -0.03452 | 0.0538 |
| Employed | **0.078925** | **0.001** | 0.03131 | 0.126539 |
| Multimorbidity |  |  |  |  |
| 2 | **-0.03975** | **0.02** | -0.07336 | -0.00614 |
| 3 | **-0.06855** | **0.001** | -0.10862 | -0.02848 |
| ≥4 | **-0.25313** | **0** | -0.39519 | -0.11106 |
|  |  |  |  |  |
| _cons | 1.389379 | 0 | 1.243375 | 1.535383 |
| /lns_1 | -1.15052 | 0 | -1.19163 | -1.10942 |
| sigma1 | 0.316471 |  | 0.303726 | 0.329751 |
| RMSE | 0.211 |  |  |  |

Reference: Male, no/primary educational level, live alone, retired, and no multimorbidity

95%C.I., 95% confidence interval; RMSE, root mean square error

Table A-6 the results of LQR

|  | beta | p-value | 95% C.I. | |
| --- | --- | --- | --- | --- |
| Female | **-0.74035** | **0** | -1.12449 | -0.3562 |
| Age | **-0.03238** | **0** | -0.05055 | -0.01421 |
| Secondary/post | 0.259051 | 0.171 | -0.11204 | 0.630144 |
| Tertiary | 0.16633 | 0.366 | -0.19465 | 0.527314 |
| Live with family/others | 0.513678 | 0.005 | 0.153201 | 0.874155 |
| Live in institution | -0.5181 | 0.436 | -1.82238 | 0.786182 |
| Unemployed | 0.222246 | 0.152 | -0.08184 | 0.52633 |
| Employed | **13.26211** | **0** | 12.45478 | 14.06944 |
| Multimorbidity |  |  |  |  |
| 2 | -0.19429 | 0.096 | -0.42278 | 0.034204 |
| 3 | **-0.37412** | **0.021** | -0.6922 | -0.05604 |
| ≥4 | **-1.52584** | **0** | -2.27482 | -0.77686 |
|  |  |  |  |  |
| RMSE | 0.82 |  |  |  |

Reference: Male, no/primary educational level, live alone, retired, and no multimorbidity

95%C.I., 95% confidence interval; RMSE, root mean square error

Table A-7 the most frequent reported health states of EQ-5D stratified by sex

| **Male** | | | | |  | **Female** | | | | |
| --- | --- | --- | --- | --- | --- | --- | --- | --- | --- | --- |
| **State** | **n** | **%** | **%%** | **Index score** |  | **State** | **n** | **%** | **%%** | **Index score** |
| 11111 | 597 | 52.23 | 52.23 | 1.0 |  | 11111 | 400 | 35 | 35 | 1.0 |
| 11121 | 156 | 13.65 | 65.88 | 0.9244 |  | 11121 | 183 | 16.01 | 51.01 | 0.9244 |
| 11122 | 28 | 2.45 | 68.33 | 0.8443 |  | 11122 | 45 | 3.94 | 54.95 | 0.8443 |
| 11112 | 25 | 2.19 | 70.52 | 0.9199 |  | 11112 | 32 | 2.8 | 57.75 | 0.9199 |
| 21221 | 16 | 1.4 | 71.92 | 0.7478 |  | 11131 | 30 | 2.62 | 60.37 | 0.8527 |
| 11131 | 14 | 1.22 | 73.14 | 0.8527 |  | 21221 | 25 | 2.19 | 62.56 | 0.7478 |
| 11221 | 14 | 1.22 | 74.36 | 0.8572 |  | 11132 | 23 | 2.01 | 64.57 | 0.7726 |
| 21222 | 13 | 1.14 | 75.5 | 0.6677 |  | 21222 | 20 | 1.75 | 66.32 | 0.6677 |
| 22222 | 13 | 1.14 | 76.64 | 0.581 |  | 11221 | 19 | 1.66 | 67.98 | 0.8572 |
| 11133 | 12 | 1.05 | 77.69 | 0.7127 |  | 22222 | 18 | 1.57 | 69.55 | 0.581 |
| 22211 | 12 | 1.05 | 78.74 | 0.7367 |  | 21121 | 14 | 1.22 | 70.77 | 0.815 |
| 21211 | 11 | 0.96 | 79.7 | 0.8234 |  | 21211 | 14 | 1.22 | 71.99 | 0.8234 |
| 22221 | 10 | 0.87 | 80.57 | 0.6611 |  | 11133 | 13 | 1.14 | 73.13 | 0.7127 |
| 11222 | 9 | 0.79 | 81.36 | 0.7771 |  | 11222 | 11 | 0.96 | 74.09 | 0.7771 |
| 21121 | 9 | 0.79 | 82.15 | 0.815 |  | 11211 | 9 | 0.79 | 74.88 | 0.9328 |
| 21111 | 7 | 0.61 | 82.76 | 0.8906 |  | 21122 | 9 | 0.79 | 75.67 | 0.7349 |
| 22322 | 7 | 0.61 | 83.37 | 0.5541 |  | 22221 | 9 | 0.79 | 76.46 | 0.6611 |
| 11113 | 6 | 0.52 | 83.89 | 0.86 |  | 32221 | 9 | 0.79 | 77.25 | 0.5882 |
| 11132 | 6 | 0.52 | 84.41 | 0.7726 |  | 11113 | 8 | 0.7 | 77.95 | 0.86 |
| 11211 | 6 | 0.52 | 84.93 | 0.9328 |  | 21232 | 8 | 0.7 | 78.65 | 0.596 |
| 22231 | 6 | 0.52 | 85.45 | 0.5894 |  | 22322 | 8 | 0.7 | 79.35 | 0.5541 |
| 22332 | 6 | 0.52 | 85.97 | 0.4824 |  | 11123 | 7 | 0.61 | 79.96 | 0.7844 |
| 21231 | 5 | 0.44 | 86.41 | 0.6761 |  | 21231 | 7 | 0.61 | 80.57 | 0.6761 |
| 22232 | 5 | 0.44 | 86.85 | 0.5093 |  | 22211 | 7 | 0.61 | 81.18 | 0.7367 |
| 31111 | 4 | 0.35 | 87.2 | 0.8177 |  | 22231 | 7 | 0.61 | 81.79 | 0.5894 |
| 11114 | 3 | 0.26 | 87.46 | 0.7069 |  | 32333 | 7 | 0.61 | 82.4 | 0.3496 |
| 11141 | 3 | 0.26 | 87.72 | 0.6926 |  | 21132 | 6 | 0.52 | 82.92 | 0.6632 |
| 11142 | 3 | 0.26 | 87.98 | 0.6125 |  | 22232 | 6 | 0.52 | 83.44 | 0.5093 |
| 21122 | 3 | 0.26 | 88.24 | 0.7349 |  | 22233 | 6 | 0.52 | 83.96 | 0.4494 |
| 21321 | 3 | 0.26 | 88.5 | 0.7209 |  | 31311 | 6 | 0.52 | 84.48 | 0.7236 |
| 22111 | 3 | 0.26 | 88.76 | 0.8039 |  | 32322 | 6 | 0.52 | 85 | 0.4812 |
| 32211 | 3 | 0.26 | 89.02 | 0.6638 |  | 21111 | 5 | 0.44 | 85.44 | 0.8906 |
| .  .  . | .  .  . | .  .  . | .  .  . | .  .  . | .  .  . | .  .  . | .  .  . | .  .  . | .  .  . | .  .  . |
| 55544 | 1 | 0.09 | 100.00 | -0.7625 |  | 55555 | 1 | 0.09 | 100.00 | -0.8637 |

For male, 131 states in total; and for female, 174 states in total

%%, cumulative percentage
